# Preoperative radiographic and histopathologic evaluation of central chondrosarcoma

**DOI:** 10.1007/s00402-013-1800-z

**Published:** 2013-07-03

**Authors:** Yasuo Yoshimura, Ken-ichi Isobe, Hideki Arai, Kaoru Aoki, Munehisa Kito, Hiroyuki Kato

**Affiliations:** Department of Orthopaedic Surgery, Shinshu University School of Medicine, 3-1-1 Asahi, Matsumoto, Nagano Prefecture Japan

**Keywords:** Chondrosarcoma, Imaging features, Histopathology, Surgical staging

## Abstract

**Background:**

Distinguishing grade 1 chondrosarcoma from grade 2 chondrosarcoma is critical both for planning the surgical procedure and for predicting the outcome. We aimed to review the preoperative radiographic and histologic findings, and to evaluate the reliability of preoperative grading.

**Methods:**

We retrospectively reviewed the medical records of 17 patients diagnosed with central chondrosarcoma at our institution between 1996 and 2011. In these cases, we compared the preoperative and postoperative histologic grades, and evaluated the reliability of the preoperative histologic grading. We also assessed the preoperative radiographic findings obtained using plain radiography, computed tomography (CT), and magnetic resonance imaging (MRI).

**Results:**

Preoperative histologic grade was 1 in 12 patients, 2 in 4 patients, and 3 in 1 patient. However, 6 of the 12 cases classified as grade 1 before surgery were re-classified as grade 2 postoperatively. In the radiographic evaluation, grade 1 was suspected by the presence of a ring-and-arc pattern of calcification on plain radiography and CT and entrapped fat and ring-and-arc enhancement on MRI. Grades 2 and 3 were suspected by the absence of calcification and the presence of cortical penetration and endosteal scalloping on plain radiography and CT, as well as soft-tissue mass formation on MRI.

**Conclusion:**

Although the combination of radiographic interpretation and histologic findings may improve the accuracy of preoperative grading in chondrosarcoma, the establishment of a standard evaluation system with the histologic and radiographic findings and/or the development of new biologic markers are necessary for preoperative discrimination of low-grade chondrosarcoma from high-grade chondrosarcoma.

## Introduction

Chondrosarcoma is the second most frequent primary malignant bone tumor after osteosarcoma [1] and represents a heterogeneous group of tumors, ranging from indolent, low-grade lesions (grade 1) to aggressive, high-grade neoplasms (grades 2, 3). Recently, some authors advocated the adequacy of intralesional surgery for grade 1 chondrosarcoma [2–8]. However, this tumor remains a challenge to diagnose accurately and treat effectively.

The histologic grading system is separated into three grades, based on cellularity, atypia, and pleomorphism [9], and is related to prognosis [10–12]. Accurate preoperative grading of chondrosarcoma is required because it determines the surgical approach and outcome. However, biopsy specimens do not always determine a correct diagnosis of grade [13, 14]. For this reason, it is particularly important to combine radiographic interpretation with histologic findings. Recent studies of magnetic resonance imaging (MRI) have improved the sensitivity in differential diagnosis [15–17]. The purpose of this study was to review the preoperative radiographic and histologic findings that differentiate low-grade chondrosarcoma (grade 1) from high-grade chondrosarcoma (grades 2, 3), and to evaluate the reliability of preoperative grading.

## Materials and methods

This study was conducted with the approval of our institutional review board.

We retrospectively reviewed the medical records of 17 patients diagnosed with central chondrosarcoma at our institution from 1 January 1996 to 31 December 2011. There were 10 men and 7 women, with a mean age at time of diagnosis of 66 years (range 38–85 years). The tumors were located in the humerus (*n* = 4), ulna (*n* = 1), phalange (*n* = 2), femur (*n* = 7), tibia (*n* = 1), calcaneus (*n* = 1), and rib (*n* = 1). The final histologic grade of the resected tumor was 1 in 6 patients, 2 in 10 patients, and 3 in 1 patient.

**Fig. 1:**
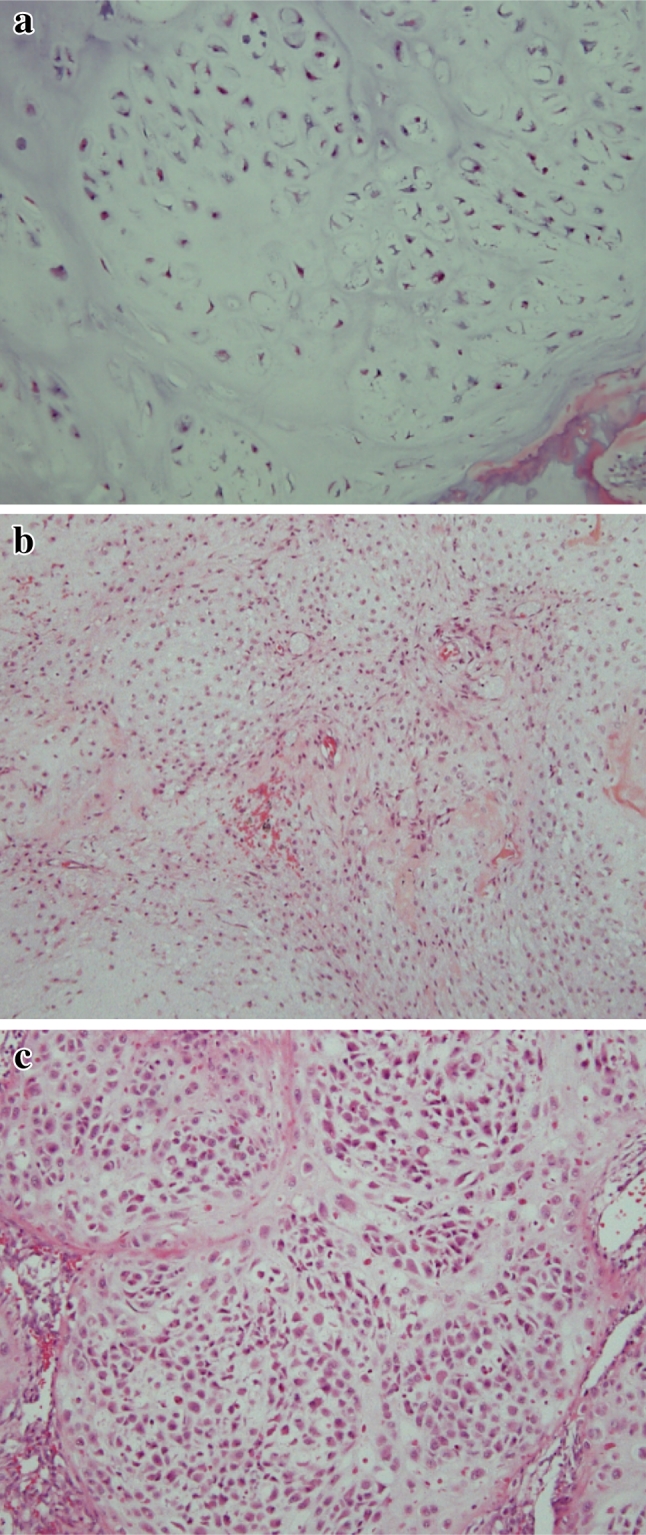
Histologic features of chondrosarcoma. **a** Grade 1: tumors are moderately cellular with chondroid matrix and absent mitosis. **b** Grade 2: tumors are more cellular with a greater degree of nuclear atypia and hyperchromasia and larger nuclear size. **c** Grade 3: tumors are densely cellular and pleomorphic. Mitoses are easily detected [hematoxylin and eosin staining; original magnification, ×100 in (**a**–**c**)]

In these cases, we compared the preoperative and postoperative histologic grading and evaluated the reliability of the preoperative histologic grading. The histologic grading system we used was based on that described by Evans and colleagues [9]. The typical histology of each grade is shown in Fig. 1. We also assessed the preoperative radiographic findings on plain radiography, computed tomography (CT), and MRI.

**Fig. 2:**
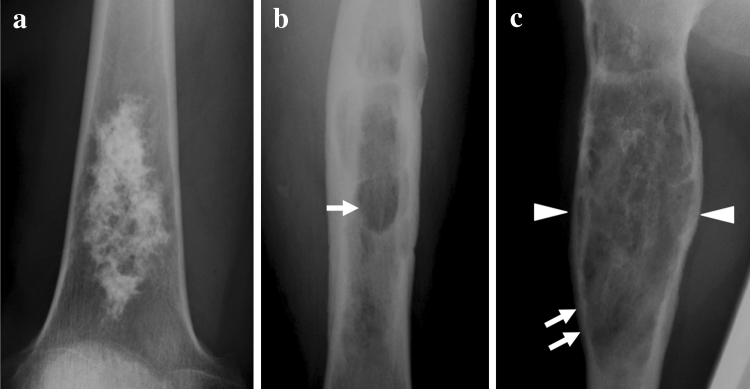
Radiographic findings of chondrosarcoma. **a** Presence of a ring-and-arc pattern of calcification. **b** Absence of calcification (*arrow*). **c** Presence of cortical penetration (*arrows*) and endosteal scalloping (*arrowheads*) is defined

**Fig. 3:**
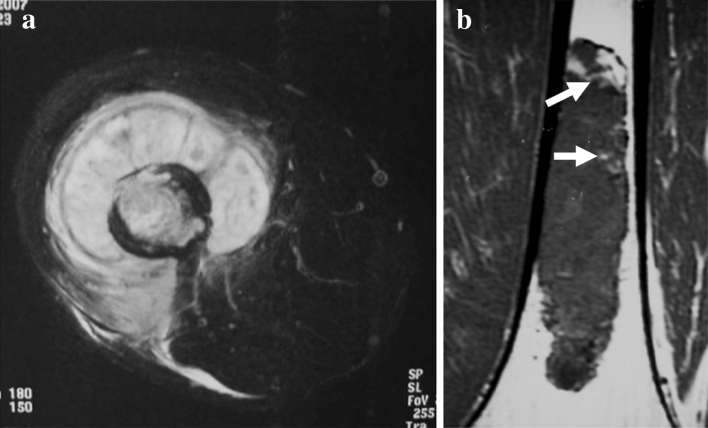
Magnetic resonance imaging findings of chondrosarcoma. **a** Axial T2-weighted fat-suppressed image showing soft-tissue mass formation. **b** Coronal T1-weighted image showing entrapped fat (*arrows*), which indicates entrapped areas of pre-existing yellow marrow

On plain radiography and CT, we evaluated the findings of low-grade chondrosarcoma (grade 1), such as a ring-and-arc pattern of calcification, and those of high-grade chondrosarcoma (grades 2, 3), such as the absence of calcification and the presence of cortical penetration and endosteal scalloping [18]. On MRI, we evaluated the findings of low-grade chondrosarcoma, such as entrapped fat within the tumor, lobular architecture, and ring-and-arc enhancement, and those of high-grade chondrosarcoma, such as central high signal on T1-weighted images, soft-tissue mass formation, and central non-enhancement portion [19]. The ring-and-arc pattern of calcification usually represents the pathologic characteristic of enchondral ossification about the margin of the cartilaginous lobules (Fig. 2a) [18]. Higher-grade chondrosarcomas often contain relatively less extensive areas of matrix mineralization. The absence of calcification and the presence of cortical penetration, endosteal scalloping, and soft-tissue mass formation represent a more aggressive pattern, which may be seen with higher-grade (grades 2, 3) chondrosarcoma (Figs. 2b, c, and 3a). Entrapped fat on T1-weighted MR images is defined as a less-aggressive finding, with entrapped areas of pre-existing yellow marrow, which is seen as small, speckled, punctate lesions (Fig. 3b). Central high signal on T1-weighted MR images and a central non-enhancement pattern represent hemorrhagic cystic change within the tumor, which may be seen in high-grade chondrosarcoma [19].

Radiographic grading was determined by these findings and compared with the postoperative histologic grading. Finally, we reviewed the reliability and limitations of this combination of preoperative histologic and radiographic gradings.

## Results

The clinical and histologic data of the 17 patients are summarized in Table 1. The method of preoperative histologic grading was needle biopsy in 4 patients, excisional biopsy in 12 patients, and curettage in 1 patient. Preoperative histologic grading was 1 in 12 patients, 2 in 4 patients, and 3 in 1 patient. However, 6 of 12 patients evaluated as grade 1 before surgery were changed to grade 2 in postoperative grading. In these 6 cases, 2 of 4 patients were diagnosed via needle biopsy. In 3 of 5 patients who had metastases, the histologic grade was changed from grade 1 to grade 2 after surgery. One of two grade 2 patients with local recurrence was evaluated as grade 1 before surgery and was selected for intralesional curettage.

**Table 1:** Clinical and histologic data of the 17 patients

No.	Age, years	Sex	Location	Biopsy	Operation	Recurrence	Metastasis	Histological grading	
								Preop	Postop
1	73	F	Femur	Excision	Wide resection	−	−	2	2
2	71	M	Femur	Excision	Amputation	−	−	2	2
3	38	M	Tibia	Excision	Amputation	−	−	2	2
4	51	M	Femur	Excision	Wide resection	−	−	1	1
5	74	F	Phalange	Needle	Marginal resection	−	+	1	2
6	57	M	Humerus	Excision	Wide resection	+	+	1	2
7	67	F	Calcaneus	Needle	Amputation	−	+	3	3
8	71	M	Humerus	Excision	Curettage	−	−	1	1
9	75	F	Ulna	Curettage	Marginal resection	+	−	1	2
10	72	F	Phalange	Excision	Amputation	−	−	1	2
11	68	M	Femur	Excision	Curettage	−	−	1	1
12	63	M	Femur	Excision	Wide resection	−	−	1	1
13	48	F	Humerus	Excision	Curettage	−	−	1	1
14	48	M	Femur	Excision	Wide resection	−	−	1	1
15	85	M	Femur	Needle	Wide resection	−	+	1	2
16	77	F	Humerus	Excision	Wide resection	−	−	1	2
17	80	M	Rib	Needle	Wide resection	−	+	2	2

**Fig. 4:**
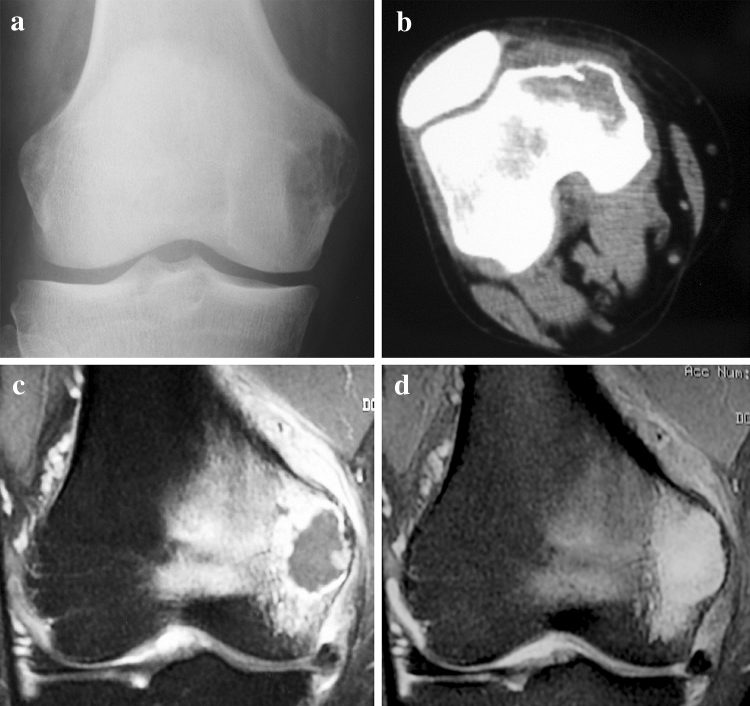
A 48-year-old man with a low-grade (grade 1) chondrosarcoma in the femur. **a**, **b** Anteroposterior radiography and computed tomography showing endosteal scalloping and the absence of calcification. **c** Coronal T1-weighted gadolinium-enhanced fat-suppressed magnetic resonance image showing a large area of central non-enhancement within the tumor. **d** Coronal T2-weighted image showing extensive bone marrow edema

In the radiographic evaluation, findings of plain radiography and CT are summarized in Table 2. Extensive areas of ring-and-arc pattern of calcification within the tumor were seen in four of six patients categorized as grade 1 (67 %) but only focally in patients of grades 2 and 3. The absence of calcification within the tumor and the presence of cortical penetration and endosteal scalloping were seen in most patients of grade 2 and 3, but only a few patients of grade 1. MRI revealed entrapped fat within the tumor on T1-weighted images and ring-and-arc enhancement on gadolinium-enhanced T1-weighted images in most patients of grade 1, while soft-tissue mass formation was seen more frequently in grades 2 and 3 (Table 3). However, in patients of grade 2, ring-and-arc enhancement was detected in only a small area of the tumor. Central high signal on T1-weighted images was seen in a few cases in all the patients. Lobular architecture and a central non-enhancing portion were detected in all grades. The results above demonstrated that grade 1 (low-grade chondrosarcoma) was suspected by the presence of an extensive area of ring-and-arc pattern of calcification on plain radiography and CT, as well as entrapped fat and ring-and-arc enhancement on MRI. However, grades 2 and 3 (high-grade chondrosarcoma) were suspected by the absence of calcification and the presence of cortical penetration and endosteal scalloping on plain radiography and CT, as well as soft-tissue mass formation on MRI (Table 4). According to these criteria, we could confirm the findings of high-grade chondrosarcoma in all six cases changed from grade 1 to grade 2 postoperatively. However, two of six cases categorized as grade 1 postoperatively had the radiographic characteristics of high-grade chondrosarcoma (Fig. 4).

**Table 2:** Radiographic features on plain radiography and computed tomography

	Grade 1, no.	Grade 2, no.	Grade 3, no.
	(*n* = 6)	(*n* = 1 0)	(*n* = 1)
Ring-and-arc pattern	4	0	0
Absence of calcification	2	10	1
Cortical penetration	2	10	1
Endosteal scalloping	1	8	1

**Table 3:** Radiographic features on magnetic resonance imaging

	Grade 1, no. (*n* = 6)	Grade 2, no. (*n* = 10)	Grade 3, no. (*n* = 1)
Entrapped fat (T1–WI)	4	1	0
Lobular architecture	5	7	1
Ring-and-arc enhancement	4	0	0
Central high signal intensity (T1–WI)	1	3	0
Soft-tissue mass formation	1	7	1
Central non-enhancement area	3	9	1

**Table 4:** Radiographic findings available for differentiation of low-grade and high-grade chondrosarcoma

	Sensitivity (%)	Specificity (%)	PPV (%)
Low grade (grade 1)			
Plain radiography and CT			
Ring-and-arc pattern	67	100	100
MRI			
Entrapped fat (T1–WI)	67	90	90
Ring-and-arc enhancement	67	100	100
High grade (grade 2)			
Plain radiography and CT			
Absence of calcification	100	67	83
Cortical penetration	100	67	83
Endosteal scalloping	80	83	89
MRI			
Soft-tissue mass formation	70	83	88

*CT* computed tomography, *MRI* magnetic resonance imaging, *PPV* positive predictive value

## Discussion

Although several studies have reviewed the differentiation criteria of grade 1 chondrosarcoma from enchondroma [13, 16, 18, 20–24], distinguishing grade 2 chondrosarcoma from grade 1 chondrosarcoma has hardly been discussed. However, distinguishing grade 1 chondrosarcoma from grade 2 chondrosarcoma is clinically among the most important aspects of diagnosis, both for planning the surgical procedure and for predicting the outcome because (1) grade 1 lesions almost never metastasize [9, 25], but grade 2 or 3 lesions tend to recur and form metastases, and (2) intralesional treatment may be adequate for grade 1 chondrosarcoma in contrast to grade 2 or 3 lesions, which may require wide resection [2–8]. We also discuss grade evaluation of several cases of chondrosarcoma at a tumor board meeting before surgery. However, the difficulties of these grade interpretations between grade 1 and grade 2 long have been suspected because there is a lack of criteria to define the limits between different subtypes. Furthermore, we often cannot determine the correct histologic grade with small biopsy specimens preoperatively since chondrosarcoma might have various histologic grades within the same tumor [11, 13]. Actually, our data indicated that preoperative histologic grading with biopsy specimens had low reliability. In our study, 6 of 12 patients evaluated as grade 1 before surgery were changed to grade 2 in postoperative grading. In these six cases, two of four patients were graded via needle biopsy. According to previous reports, a needle biopsy does not always determine a correct diagnosis of grade [12, 15]. Although needle biopsy may be performed as the first method for histologic evaluation, excisional biopsy should be performed as well when the differentiation of grade 1 from grade 2 chondrosarcoma is difficult by needle biopsy alone.

Therefore, it is particularly important to combine radiographic interpretation with histologic findings. In our study, the presence of an extensive area of ring-and-arc pattern of calcification is more common in low-grade (grade 1) chondrosarcoma, while the absence of calcification and the presence of cortical penetration and endosteal scalloping is more common in high-grade (grades 2, 3) chondrosarcoma on plain radiography and CT. Moreover, entrapped fat within the tumor and ring-and-arc enhancement are more characteristic of low-grade chondrosarcoma, but soft-tissue mass formation is more common in high-grade chondrosarcoma on MRI. Taken together, our data indicated that an extensive area of ring-and-arc pattern of calcification on CT and entrapped fat within the tumor and ring-and-arc enhancement on MRI had intermediate sensitivity (67, 67, 67 %, respectively), high specificity (100, 90, 100 %, respectively), and a positive predictive value (PPV) (100, 90, 100 %, respectively) in grade 1 chondrosarcoma. The absence of calcification and the presence of cortical penetration and endosteal scalloping on plain radiography and CT had high sensitivity (100, 100, 80 %, respectively) and a PPV (83, 83, 89 %, respectively), while soft-tissue mass formation on MRI had high specificity (83 %) and a PPV (88 %) in grade 2 chondrosarcoma (Table 4).

A previous study reported that a relatively less extensive area of matrix mineralization is often seen in higher-grade chondrosarcomas on plain radiography and CT [18]. Another study reported that soft-tissue mass formation favors the diagnosis of high-grade chondrosarcoma, and entrapped fat within the tumor is highly indicative of low-grade chondrosarcoma on MRI [19]. In addition, the radiographic classification of Lodwick is used as an index to predict the biologic behavior of bone tumors and tumor-like lesions; this classification is commonly applied for chondrosarcoma [26, 27]. Our data, which were gathered by evaluating plain radiography, CT, and MRI in the same series, were consistent with the results of previous studies. Therefore, we advocate preoperative grading in the context of a combination of findings on plain radiography, CT, and MRI to more accurately differentiate low-grade chondrosarcoma from high-grade chondrosarcoma since some cases do not have characteristic radiographic findings. If treating physicians use this method, they can avoid treating as grade 1 for grade 2 chondrosarcoma. However, two cases of grade 1 chondrosarcoma that were evaluated as grade 2 preoperatively with radiographic findings were treated with wide resection in the present study. Thus, the value of morphologic radiographic features in differentiating low-grade chondrosarcoma from high-grade chondrosarcoma was shown to be limited. In the present status, we should select wide resection if the radiographic findings have the characteristics of grade 2 in spite of a histologic evaluation pointing to grade 1. In addition, it is necessary to consider the location of the chondrosarcoma when determining the treatment strategy because chondrosarcomas arising from the pelvis or axial skeleton are generally associated with greater biologic aggressiveness and more frequent recurrence. Wide resection should be selected for these locations even if the radiographic and/or histologic findings show characteristics of grade 1 chondrosarcoma.

Recently, a new biomarker has been investigated for its ability to distinguish low-grade from high-grade chondrosarcoma and to predict clinical outcomes. Takeuchi et al. [28] demonstrated that significant differences in the endogenous secretory receptor for advanced glycation end products (esRAGE) labeling index were seen between grade 1 and grade 2 chondrosarcomas using an enzyme-linked immunosorbent assay (ELISA) in 11 cases of chondrosarcoma. However, further large-scale prospective studies are required to validate these markers as routine diagnostic and prognostic tools in the assessment of chondrosarcoma. In the future, the establishment of a standard evaluation system combining radiographic interpretation with histologic findings and/or the development of new biologic markers is necessary for accurately discriminating low-grade chondrosarcoma from high-grade chondrosarcoma prior to surgery.
